# Completely conserved VP2 residue K140 of KREMEN1-dependent enteroviruses is critical for virus-receptor interactions and viral infection

**DOI:** 10.1128/mbio.03040-24

**Published:** 2025-01-16

**Authors:** Zeyu Liu, Xue Li, Xiaohong Li, Xingyu Yan, Yuan Tian, Yue Zhao, Kexin Liu, Pei Hao, Shuye Zhang, Chao Zhang

**Affiliations:** 1 Shanghai Institute of Infectious Disease and Biosecurity, Fudan University 12478 , Shanghai, China; 2 Shanghai Institute of Immunity and Infection, Chinese Academy of Sciences, University of Chinese Academy of Sciences, Shanghai, China; 3 Clinical Center for Biotherapy, Zhongshan Hospital, Fudan University 92323 , Shanghai, China; 4 Institutional Center for Shared Technologies and Facilities of Shanghai Institute of Immunity and Infection, Chinese Academy of Sciences, Shanghai, China; Duke University School of Medicine, Durham, North Carolina, USA

**Keywords:** hand, foot, and mouth disease, enterovirus, KREMEN1 receptor, conservation

## Abstract

The KREMEN1 (KRM1) protein is a cellular receptor for multiple enteroviruses that cause hand, foot, and mouth disease (HFMD), including coxsackievirus CVA2, CVA3, CVA4, CVA5, CVA6, CVA10, and CVA12. The molecular basis for the broad recognition of these viruses by the KRM1 receptor remains unclear. Here, we report the indispensable role of the completely conserved VP2 capsid protein residue K140 (designated K2140) in mediating receptor recognition and infection by CVA10 and other KRM1-dependent enteroviruses. Residue K2140 not only facilitates receptor recognition, cell attachment, and infection of CVA10 but also contributes to CVA10 pathogenicity *in vivo*. Notably, residue K2140 is completely conserved in all strains of the KRM1-dependent enteroviruses. Mutational analysis confirms the importance of K2140 for infection by CVA2–CVA6, and CVA12. Moreover, CVA8, an enterovirus for which the cellular receptor has not yet been identified, also possesses the conserved K2140 residue. We experimentally demonstrate that CVA8 utilizes KRM1 as its receptor, with K2140 being essential for viral infection. Additionally, residue D90 of KRM1 engages with residue K2140 and plays a crucial role in KRM1-mediated enterovirus infections. Collectively, our findings underscore the significance of the absolutely conserved K2140 residue in receptor interactions and infection of all KRM1-binding enteroviruses, providing novel insights into the molecular basis of enterovirus infection and informing the development of broad-spectrum therapies against HFMD.

**IMPORTANCE:**

Hand, foot, and mouth disease (HFMD) annually affects millions of children worldwide. HFMD is caused by various enteroviruses, such as coxsackieviruses CVA6, CVA16, CVA10, and enterovirus 71 (EV-A71). Licensed inactivated EV-A71 vaccines do not provide cross-protection against other enteroviruses. There are no drugs specifically for HFMD. KREMEN1 (KRM1) serves as the cellular receptor for many HFMD-related enteroviruses, including CVA2–CVA6, CVA10, and CVA12. However, the molecular basis for broad recognition of these enteroviruses by the KRM1 receptor remains elusive. Here, we report that VP2 residue K140 (K2140) is completely conserved among all KRM1-dependent enteroviruses and is essential for virus-receptor binding and viral infection by interacting with residue D90 of KRM1. Overall, our findings provide a deeper understanding of the molecular basis of KRM1-dependent enterovirus infection *in vitro* and *in vivo* and may contribute to the development of broad-spectrum anti-enterovirus vaccines and treatments.

## INTRODUCTION

Species A enteroviruses (EV-A) in the genus *Enterovirus* consist of 25 serotypes: coxsackievirus CVA2–A8, CVA10, CVA12, CVA14, CVA16, and enterovirus EV-A71, EV-A76, EV-A89 to -A92, EV-A114, and EV-A119 to -A125 (1, 2). EV-A92 and EV-A122 to -A125 (formerly simian virus 19, 43, and 46 and baboon enterovirus A13) are non-human enteroviruses (1, 2). EV-A infections cause a wide spectrum of diseases, including hand, foot, and mouth disease (HFMD), herpangina, encephalitis, aseptic meningitis, and acute flaccid paralysis (3). HFMD is a common childhood illness, characterized by typical manifestations such as fever, mouth sores, and rash on the hands and feet. HFMD is usually mild and self-limited. However, severe forms of HFMD can lead to death or long-term sequelae due to neurological complications and cardiopulmonary failure (4–6). In the past, most HFMD cases were caused by EV-A71 and CVA16. However, in recent years, outbreaks of CVA6- and CVA10-associated HFMD have significantly increased worldwide, especially in China (7, 8). Currently, the sole licensed vaccine for HFMD prevention is the EV-A71 inactivated vaccine, which confers protection against EV-A71 but not against other enteroviruses (9).

**Fig 1:**
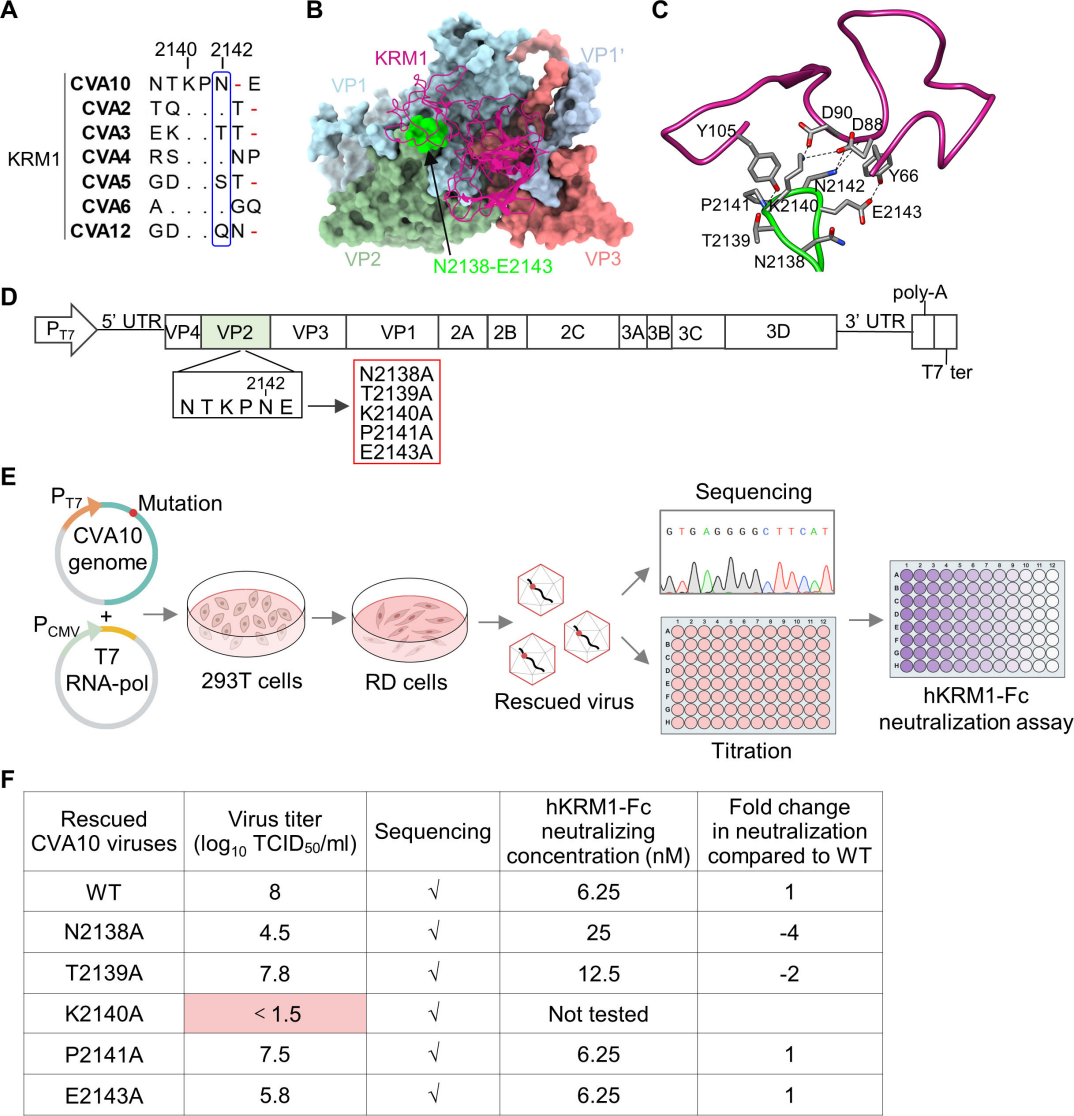
VP2 residue K140 is essential for the rescue of CVA10. (**A**) Alignment (part) of amino acid sequences of VP2 EF loop of the KRM1-using enteroviruses. VP2 residues are numbered starting from 2001. The residue 2142 is boxed. (**B and C**) The residues N2138–E2143 at the tip of VP2 EF loop of CVA10 have close contact with the KRM1 receptor (PDB: 7BZU). In panel B, KRM1 is colored medium violet red and shown in cartoon representation. One asymmetric unit of CVA10 is shown as a surface representation. VP1, light blue; VP2, dark sea green; VP3, light coral. VP1’, the C terminus of an adjacent VP1 is colored in light steel blue. Residues N2138–E2143 are colored in lime. In panel C, interactions of KRM1 with VP2 EF loop. KRM1, violet red. VP2-EF loop, green. Nitrogen atom, blue; oxygen, red; carbon, gray. Black dashed lines indicate hydrogen bonds. Residue K2140 of CVA10 also forms salt bridges with residues D88 and D90 of KRM1. (**D**) Schematic representation of the construction of infectious clones of CVA10 mutants. P_T7_, T7 promoter. (**E**) Schematic of recovery and characterization of mutant CVA10 viruses. T7 RNA-pol, and T7 RNA polymerase. (**F**) Rescued wild-type (WT) and mutant CVA10 viruses were titrated by TCID_50_ assay and tested for resistance to neutralization with soluble hKRM1-Fc. Fold increase or decrease (prefixed with a minus sign) in neutralization concentration of hKRM1-Fc against the mutants with respect to wild-type CVA10 was calculated and shown.

Enteroviruses are non-enveloped, single-stranded RNA viruses with icosahedral capsids composed of 60 copies of each of four capsid proteins, VP1, VP2, VP3, and internal VP4 (3). On the enterovirus surface, there is a prominent star-shaped plateau (mesa) at the 5-fold axis, surrounded by a deep depression (termed the canyon), and a propeller-like protrusion at the 3-fold axis (10–14). EV-A71, CVA16, CVA7, and CVA14 use SCARB2 as the main receptor (15, 16). CVA2, CVA3, CVA4, CVA5, CVA6, CVA10, and CVA12 have been reported to use KREMEN1 (KRM1) protein as a functional cellular receptor (17). Although structural analysis revealed that KRM1 receptor binds to the canyon region of CVA10 virion (12, 14), the molecular basis for the broad recognition of a variety of enteroviruses by KRM1 receptor remains unclear. We previously reported that VP2 residue N142 (designated N2142) is crucial for KRM1 receptor binding and infection of CVA10 (18). However, N2142 is highly conserved in CVA2, CVA4, and CVA6, but not in CVA3, CVA5, and CVA12, which have other amino acids (T, S, or Q) at this position (Fig. 1A). Thus, N2142 residue alone is not sufficient to explain why a range of enteroviruses can utilize the same KRM1 receptor protein.

In this study, we identified VP2 residue K140 (designated K2140) as a crucial residue for CVA10 infection by scanning mutagenesis of KRM1 receptor-contacting residues. K2140 plays a vital role not only in receptor recognition, cell attachment, and infection of CVA10 but also in CVA10 virulence in mice. Despite its prominent position on the virion surface, K2140 is completely conserved among all KRM1-dependent enteroviruses, underscoring its importance in viral infections. K2140 interacts with residue D90 of KRM1, facilitating viral infection. In addition, CVA8, another member of EV-A, also possesses K2140 and can utilize KRM1 as its receptor. In summary, our findings highlight the importance of the fully conserved K2140 residue in receptor interactions and infections of all KRM1-binding enteroviruses, offering novel insights into the molecular mechanisms of enterovirus infection and informing the development of broad-spectrum anti-HFMD drugs.

## RESULTS

### VP2 residue K140 is essential for the rescue of CVA10

We previously reported that VP2 residue N142 (designated N2142) of CVA10 is critical for KRM1 receptor binding and viral infection (18). Note that VP2 residues are numbered starting from 2001. Residue N2142 is situated in the highly surface-exposed VP2 EF loop. Other residues at the tip of CVA10 VP2 EF loop, including N2138, T2139, K2140, P2141, and E2143, also have close contact with the KRM1 receptor (Fig. 1B through C), revealed by the previously reported cryo-electron microscopy (cryo-EM) structure of mature CVA10 in complex with KRM1 at pH 5.5 (PDB: 7BZU) (12, 14). We wanted to investigate whether these residues also play a role in CVA10 infection and the CVA10-KRM1 interaction.

To this end, N2138A, T2139A, K2140A, P2141A, and E2143A mutations were separately introduced into the previously constructed infectious clone of CVA10 prototype strain Kowalik (18), which was driven by T7 promoter (Fig. 1D). To rescue CVA10 viruses, these infectious clone plasmids were co-transfected into HEK 293T cells with the T7 RNA polymerase (RNA-pol) over-expression plasmid. The rescued viruses were amplified in RD cells, sequenced, titrated via TCID_50_ assay, and tested for the sensitivity to neutralization by soluble hKRM1-Fc protein (human IgG Fc-fused human KRM1 ectodomain) (18) (Fig. 1E). CVA10 viruses WT (wild type), N2138A, T2139A, P2141A, and E2143A were successfully rescued and confirmed by Sanger sequencing (Fig. 1F). Moreover, we found that the rescued N2138A, T2139A, P2141A, and E2143A mutants were still sensitive to neutralization with hKRM1-Fc, compared with the rescued CVA10-WT (Fig. 1F). For the K2140A mutant, no obvious cytopathic effect (CPE) was observed in RD cell culture, and its infectious viral titer was below the detection limit (10^1.5^ TCID_50_/mL) (Fig. 1F). Thus, the K2140 residue is essential for the rescue of CVA10, whereas the N2138, T2139, P2141, and E2143 residues are not.

### CVA10 residue K2140 is critical for KRM1 receptor binding, viral attachment, and infection of RD cells

**Fig 2:**
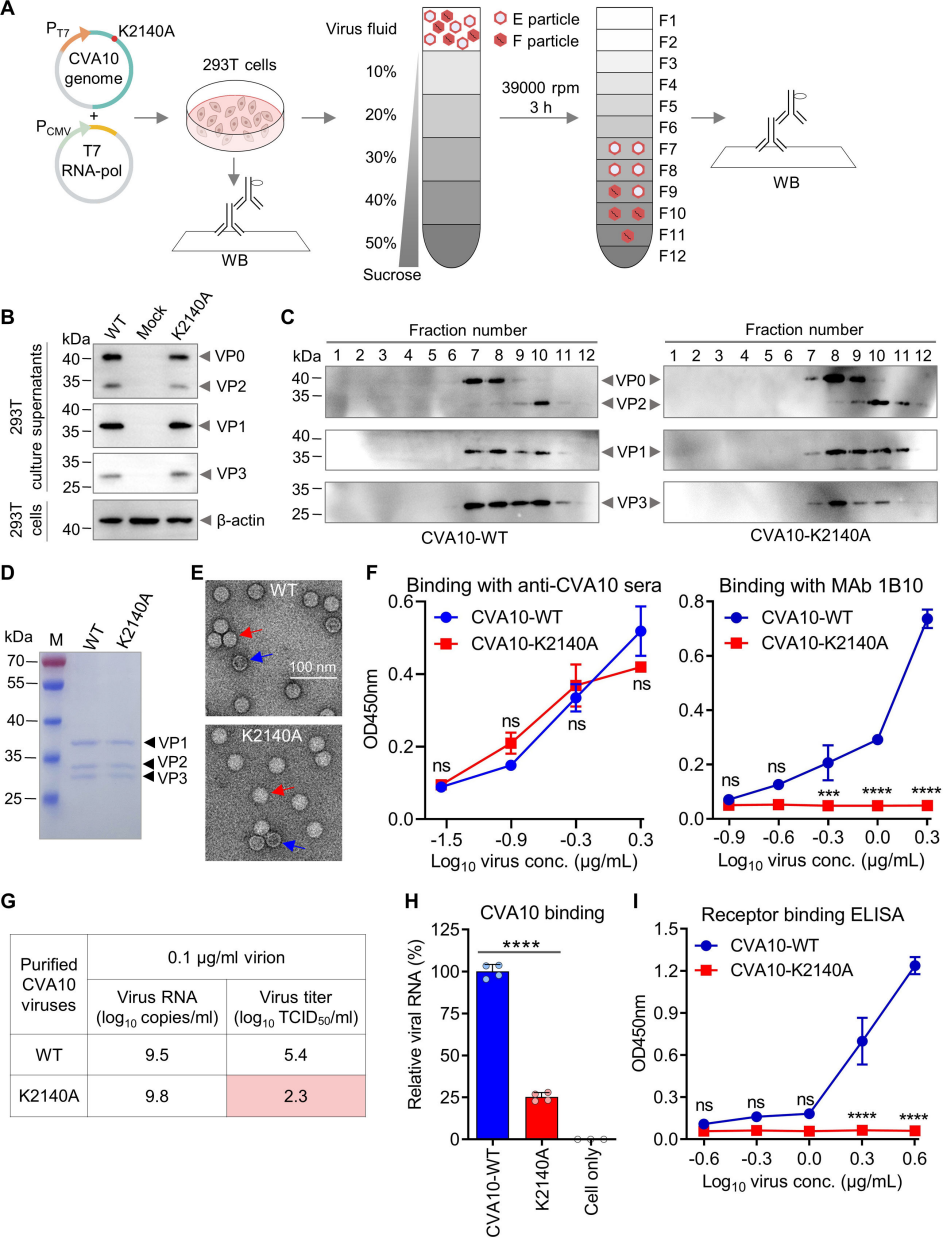
K2140A mutation could reduce viral infection and attachment by impairing KRM1 receptor binding. (**A**) Schematic of recovery and characterization of CVA10-K2140A mutant. WB, western blot. F1, fraction #1. E particle, empty particle. F particle, full particle (mature virion). (**B**) Western blotting analysis of culture supernatants from transfected 293T cells with anti-VP0, anti-VP1, and anti-VP3 polyclonal antibodies. Cell lysates were analyzed by western blotting with an anti-β-actin antibody. Mock, 293T cells transfected with the empty vector pVAX1 and the T7 RNA-pol plasmid. Note that 16 µL of 293T cell culture supernatants or lysates from 1  ×  10^5^ cells were boiled in an SDS loading buffer and loaded onto the gel. (**C**) Sucrose gradient analyses. Supernatants (700 µL) from transfected 293T cells were subjected to 10%–50% sucrose gradient centrifugation. Twelve fractions (350 µL/fraction) were collected and analyzed by western blotting with anti-VP0, anti-VP1, and anti-VP3 polyclonal antibodies. Sixteen microliters of gradient fractions were boiled and loaded onto the gel. (**D**) SDS-PAGE analysis of purified CVA10-WT and CVA10-K2140A. M, protein marker. In total, 1.5 µg of total protein per sample was loaded onto the gel. (**E**) Electron microscopy of purified CVA10-WT and CVA10-K2140A after negative staining. Bar = 100 nm. Red and blue arrows indicate mature virion and empty capsid, respectively. (**F**) The reactivities of anti-CVA10 sera and neutralizing MAb 1B10 to purified CVA10-WT and CVA10-K2140A viral particles were determined by ELISA. Serially diluted viral antigens were coated onto ELISA plates, followed by incubation with anti-CVA10 antibodies. Data are mean ± SEM of triplicate wells. Conc., concentration. (**G**) Purified CVA10-WT and CVA10-K2140A viruses were diluted to 0.1 µg/mL and analyzed for viral RNA copy numbers by RT-qPCR and virus titers by TCID_50_ assay. The CVA10 infectious clone plasmid served as a standard to determine the absolute viral genome copy numbers. (**H**) Analysis of the binding of CVA10-WT and CVA10-K2140A to RD cells using RT-qPCR. 0.1 µg/mL of purified CVA10 viral particles were allowed to bind to prechilled RD cells for 2 h at 4°C, and after washes, CVA10 viral RNA levels were measured by RT-qPCR and normalized to β-actin. Data are means  ±  SD of three or four biological replicate samples. A two-tailed unpaired *t*-test was used to compare differences. ****, *P* < 0.0001. Each data point represents a separate well in a 24-well plate, corresponding to one biological replicate. (**I**) The reactivities of purified CVA10-WT and CVA10-K2140A with human KRM1-Fc protein were measured by ELISA. Serially diluted viral antigens were coated onto ELISA plates, followed by incubation with KRM1-Fc protein. Data are means  ±  SD of triplicate wells. For panels F and I, statistical analysis was performed using two-way ANOVA. ns, no significant difference (*P* ≥ 0.05); ***, *P* < 0.001; ****, *P* < 0.0001. Note that the above experiments were repeated twice with similar results.

The life cycle of enteroviruses generally consists of attachment, entry, uncoating, replication, assembly, and virion release. The K2140A mutation appears to be detrimental for CVA10 (Fig. 1F). Since residue K2140 is located in the highly exposed loop on the virus surface (Fig. 1B through C), mutation at this position is likely to affect viral attachment and entry rather than viral replication, assembly, and release. Accordingly, we have formulated a hypothesis that the K2140A mutant has been rescued in transfected 293T cells, but this mutant virus may not be able to efficiently bind to and infect RD cells. To test the hypothesis, the pVAX empty vector (mock) or the CVA10 infectious clone plasmids were transfected into HEK 293T cells together with the T7 RNA-pol expression plasmid. The 293T culture supernatants were collected at 48 h post-transfection and analyzed for viral capsid proteins by western blotting with CVA10 VP0-, VP1-, and VP3-specific antibodies (Fig. 2A). Lysates from 293T cells, rather than culture supernatants, were used for the detection of β-actin. As shown in Fig. 2B and Fig. S1A, CVA10-specific bands between 25 KD and 40 KD, representing VP0, VP2, VP1, and VP3 subunit proteins, were detected in the culture supernatants from 293T cells transfected with CVA10-WT or CVA10-K2140A infectious clone plasmids, whereas no specific bands were observed for culture supernatant from mock-transfected 293T cells.

To assess viral particle assembly, culture supernatants from transfected 293T cells were subjected to sucrose gradient ultracentrifugation, and the resulting gradient fractions were analyzed for the distribution of CVA10 capsid proteins by western blotting (Fig. 2A). For the CVA10-WT or CVA10-K2140A samples, VP0, VP2, VP1, and VP3 proteins co-migrated among fractions #7 to #11 (Fig. 2C; Fig. S1B), suggesting that these capsid proteins co-assembled into viral particles (empty particle or mature virion). Note that CVA10 empty particles consist of VP0, VP1, and VP3 proteins, whereas CVA10 mature virion is composed of VP2, VP4, VP1, and VP3 proteins (11). Taken together, these results indicate that CVA10-WT and CVA10-K2140A viral particles can be assembled and released into culture supernatants from CVA10 infectious clone-transfected 293T cells.

CVA10-WT and CVA10-K2140A samples were purified from 1 L of culture supernatants of transfected 293T cells using PEG precipitation and sucrose gradient ultracentrifugation. SDS-PAGE analysis showed that purified CVA10-WT and CVA10-K2140A viral particles mainly consisted of VP1, VP2, and VP3 proteins, corresponding to mature virion (Fig. 2D; Fig. S2A). Negative stain electron microscopy (EM) analysis confirmed that the majority of both purified CVA10-WT and CVA10-K2140A virus particles were full particles (mature virions) (Fig. 2E). After careful observation and calculation, we determined that the purified CVA10-WT sample contained 11.6% empty particles, whereas the purified CVA10-K2140A sample contained 11.4%. Therefore, the ratio of mature virions to empty particles in both purified samples is quite similar (about 7.7:1).

To further characterize the purified virus samples, CVA10-WT and CVA10-K2140A were tested by ELISA for reactivity with anti-CVA10 polyclonal mouse sera and a CVA10-specific neutralizing monoclonal antibody (MAb) called 1B10. Both CVA10-WT and CVA10-K2140A viral particles efficiently reacted with anti-CVA10 polyclonal antibody in a virus dose-dependent fashion, and their sera-binding activity levels were comparable (Fig. 2F). These results indicate that CVA10-K2140A viral particles may have a similar overall conformation to CVA10-WT. In addition, CVA10-WT bound efficiently with neutralizing MAb 1B10 in a virus dose-dependent manner, but CVA10-K2140A failed to bind 1B10 even at very high concentrations (Fig. 2F), suggesting that there are some differences between the two viruses in terms of the epitope of MAb 1B10.

We next compared the differences between CVA10-WT and CVA10-K2140A in terms of viral infection. The purification process resulted in yields of 124 µg of CVA10-WT and 90 µg of CVA10-K2140A mature virions per liter of 293T cell culture supernatants. Both purified viruses were diluted to 0.1 µg/mL and analyzed for viral RNA copy numbers by reverse transcription-quantitative PCR (RT-qPCR) and virus titers by TCID_50_ assay (Fig. 2G). Purified CVA10-WT and CVA10-K2140A viral particles at 0.1 µg/mL had comparable numbers of virus genomic RNA (10^9.5^ and 10^9.8^ copies/mL, respectively). However, the viral titer of CVA10-K2140A was determined to be 10^2.3^ TCID_50_/mL, which was 1,259-fold lower than that of CVA10-WT (10^5.4^ TCID_50_/mL). Thus, K2140A mutation resulted in a remarkable reduction in CVA10 infection in RD cells.

Attachment to the cell surface is the first step in the infection of enteroviruses. To compare the cell-binding ability between CVA10-WT and CVA10-K2140A, the same number (100 ng/mL) of purified CVA10 viral particles were allowed to bind RD cells at 4°C for 2 h, and after washing, cells were harvested for analysis of viral RNA levels by RT-qPCR. As shown in Fig. 2H, statistically significant differences in viral binding were observed between CVA10-WT and CVA10-K2140A. Specifically, the number of viral particles that bound to RD cells was reduced by 75% because of K2140A mutation, compared with CVA10-WT.

We then compared receptor (KRM1) binding capacity between CVA10-WT and CVA10-K2140A by ELISA. Serially diluted purified CVA10 viral particles were coated onto ELISA plates and then allowed to react with hKRM1-Fc protein (Fig. 2I). CVA10-WT effectively bind with hKRM1-Fc dose-dependently, whereas CVA10-K2140A failed to bind hKRM1-Fc even at a very high virus concentration (4 µg/mL). Thus, the K2140A mutation may abolish KRM1 receptor-binding activity of CVA10, consistent with the results of the viral binding assay (Fig. 2H). Taken together, these results suggest that CVA10-K2140A mutant virus cannot effectively bind to and infect RD cells because of the loss of KRM1 receptor binding activity.

### Residue K2140 is critical for CVA10 pathogenicity in mice

**Fig 3:**
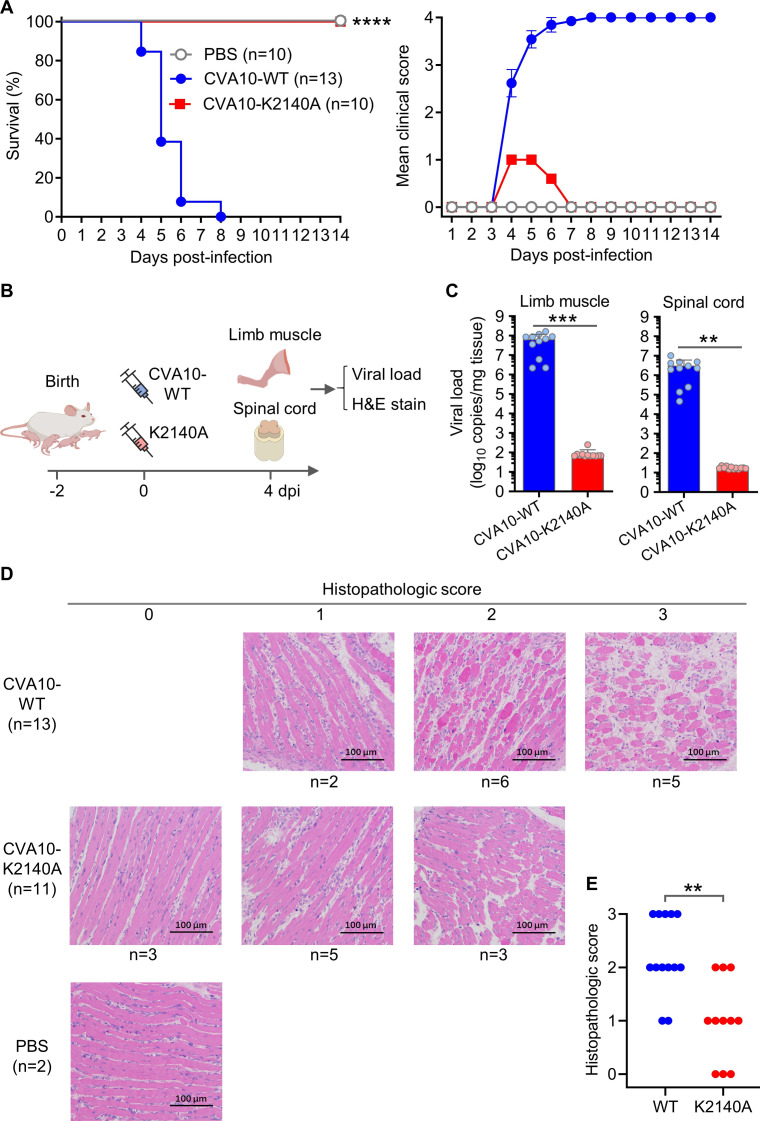
K2140A mutation could reduce the severity of CVA10-induced disease in mice. (**A**) Groups of 2-day-old ICR mice were inoculated intraperitoneally (i.p.) with PBS or 10^−5^ ng/mouse of purified CVA10-WT or CVA10-K2140A, and then survival and clinical signs were observed daily. Clinical scores were graded as follows: 0, healthy; 1, reduced mobility; 2, limb weakness; 3, limb paralysis; and 4, death. Number of mice per group were indicated in brackets. Statistical analysis of survival was assessed by log-rank (Mantel-Cox) test. ****, *P* < 0.0001. All error bars represent SEM. (**B**) Schematic showing the timing of viral load and histological analysis. (**C**) Groups of ICR mice were infected with purified CVA10-WT or CVA10-K2140A, and viral loads in limb muscle and spinal cord were determined by RT-qPCR at 4 dpi. Each symbol represents an individual mouse. For qPCR data analysis, non-detects were handled by setting undetermined values to a maximum ct (35). Data are means  ±  SD. A two-tailed unpaired *t*-test was used to compare differences. **, *P* < 0.01; ***, *P* < 0.001. (**D**) Groups of ICR mice were inoculated with PBS (control), purified CVA10-WT, or CVA10-K2140A, and histological analyses of limb muscle tissues were performed by staining sections with H&E at 4 dpi. According to the severity of myositis, histopathologic scores were graded as: 0, not present; 1, mild; 2, moderate; and 3, severe. Number of mice (N) is shown. Note that the data shown in panels c and d were generated from two different mouse experiments. (**E**) Histopathologic scores of the CVA10-WT and CVA10-K2140A groups. Each symbol represents an individual mouse. The Mann–Whitney test was used to compare differences. **, *P* < 0.01.

To determine the effect of the K2140A mutation on CVA10 pathogenesis, the same amount (10^−5^ ng/mouse) of purified rescued CVA10-WT or CVA10-K2140A viral particles were used to infect 2-day-old ICR mice. Following infection, mice were observed daily for clinical symptoms and mortality. As shown in Fig. 3A, mice infected with CVA10-WT developed clinical symptoms, including limb weakness and paralysis, and ultimately, all died within 8 days post-infection (dpi). By contrast, all mice in the CVA10-K2140A group survived, although five of them (50%) showed only transient and mild signs of disease. The results indicate that the K2140A mutation can significantly decrease the virulence of CVA10 in the mouse model.

To investigate the replication efficiency of CVA10-WT or CVA10-K2140A in mice, groups of infected mice were euthanized at 4 dpi, and limb muscle and spinal cord (the major sites of CVA10 replication) were harvested and evaluated for viral RNA levels by RT-qPCR (Fig. 3B). Note that in this study, viral loads in tissues were measured by the determination of absolute viral genome copy number via RT-qPCR, but not infectious virus titers via TCID_50_ assay, because CVA10-K2140A cannot effectively infect RD cells (Fig. 2G). As shown in Fig. 3C, viral genome copy numbers per mg of limb muscle tissues from the CVA10-WT group ranged from 2.2 × 10^6^ to 1.7 × 10^8^ (geometric mean = 3.3 × 10^7^), whereas those in the CVA10-K2140A group ranged from undetectable to 252 (geometric mean = 79). Additionally, viral genome copy numbers per mg of spinal cord tissues from the CVA10-WT group ranged from 4.6 × 10^4^ to 1.0 × 10^7^ (geometric mean = 1.4 × 10^6^), whereas those in the CVA10-K2140A group ranged from undetectable to 23 (geometric mean = 18). There were highly significant differences in viral loads in limb muscle and spinal cord tissues between the CVA10-WT and CVA10-K2140A groups. Therefore, the K2140A mutation can significantly reduce tissue viral loads of CVA10-infected mice. One can infer from Fig. 3A through C that the K2140A mutation could reduce CVA10 lethality in mice by decreasing viral loads in tissues.

To determine histopathological changes associated with CVA10-WT and CVA10-K2140A infection, limb muscle samples from CVA10-infected mice, or PBS-injected mice (control) were harvested at 4 dpi and subjected to hematoxylin and eosin (H&E) staining and histopathologic examination. Severity of myositis was graded using the following scale: 0, not present; 1, mild; 2, moderate; and 3, severe. As shown in Fig. 3D, all mice in the CVA10-WT group had mild to severe myofiber necrosis. For the CVA10-K2140A group, three of 11 infected mice showed normal muscle morphology and the other eight mice displayed mild to moderate myofiber necrosis. The CVA10-K2140A group had a significantly lower mean histopathologic score compared with the CVA10-WT group (Fig. 3E), consistent with the results of viral load determination (Fig. 3C). Thus, the K2140A mutation could reduce the extent of the muscle damage following CVA10 infection.

### A saturation-mutagenesis analysis reveals the importance of the Lys residue at position 2140 in CVA10 infection

**Fig 4:**
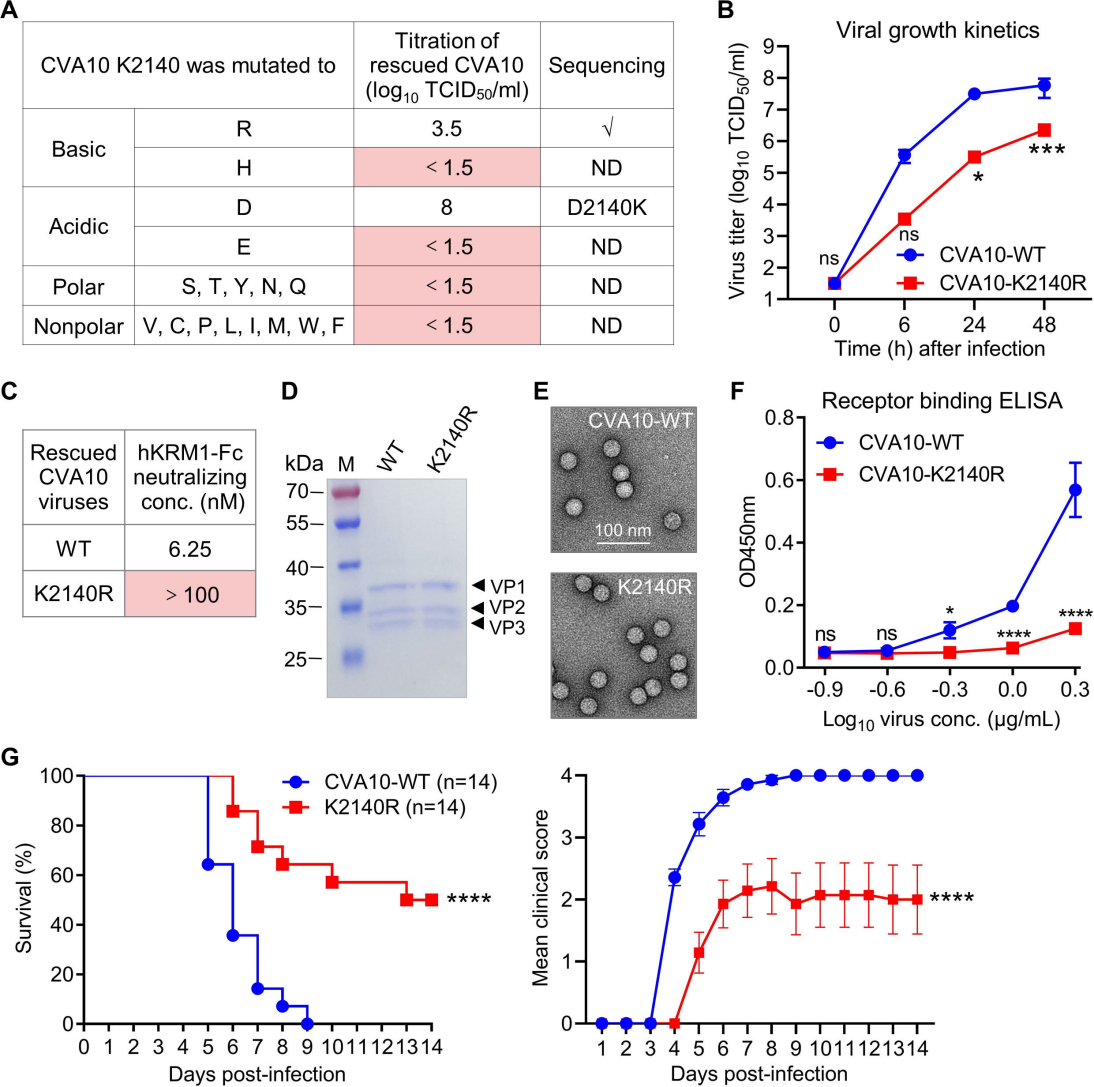
Saturation mutagenesis reveals the importance of the Lys residue at position 2,140 in CVA10 infection. (**A**) K2140 was mutated to the other amino acids, and the mutant CVA10 viruses were rescued by transfecting HEK 293T cells and amplified in RD cells, followed by titration by TCID_50_ assay and sequencing. ND, not done. (**B**) Growth kinetics of rescued CVA10-WT and CVA10-K2140R. RD cells were infected at a MOI of 0.01 and viral titers were determined at 0, 6, 24, and 48 h post-infection. Data are means  ±  SD of three biological replicate samples. Statistical analysis was assessed using two-way ANOVA. ns, no significant difference; *, *P* < 0.05; ***, *P* < 0.001. (**C**) The rescued CVA10-K2140R was tested for resistance to neutralization with KRM1-Fc. (**D**) SDS-PAGE analysis of purified CVA10-WT and CVA10-K2140R. M, protein marker. In total, 1.5 µg of total protein per sample was loaded onto the gel. (**E**) Electron microscopy of purified CVA10-WT and CVA10-K2140R after negative staining. Bar = 100 nm. (**F**) The reactivities of purified CVA10-WT and CVA10-K2140R with hKRM1-Fc protein were determined by ELISA. ELISA plates were coated with serially diluted viral antigens and then incubated with KRM1-Fc protein. Data are means  ±  SD of triplicate wells. Statistical analysis was performed using two-way ANOVA. ns, no significant difference (*P* ≥ 0.05); *, *P* < 0.05; ****, *P* < 0.0001. (**G**) Groups of 2-day-old ICR mice were infected i.p. with 1 ng/mouse of purified CVA10-WT or CVA10-K2140R, and then survival and clinical signs were observed daily. Clinical scores were graded as described in the legend of Fig. 3. Statistical analysis of survival was assessed by log-rank (Mantel-Cox) test. Statistical analysis of clinical scores was performed using two-way ANOVA. ****, *P* < 0.0001. Error bars represent SEM.

The above data demonstrate that the K2140A mutation can significantly reduce CVA10 infection *in vitro* and *in vivo* (Fig. 2 and 3). To further investigate whether the Lys residue at position 2,140 is essential for CVA10 infection, a saturation-mutagenesis analysis was performed on the CVA10 infectious clone plasmid by substituting K2140 with the other amino acids (Fig. 4A). The mutant CVA10 viruses were obtained by transfecting HEK 293T cells with the mutant infectious clone plasmids and the T7 RNA-pol plasmid and then amplified in RD cells, followed by titration by TCID_50_ assay and sequencing. As shown in Fig. 4A, only CVA10-K2140R mutant virus was successfully rescued and was genetically stable, although its infectious viral titer was relatively low. We failed to rescue CVA10-K2140D mutant because reverse mutation occurred in cell culture. In addition, attempts to rescue and amplify the other CVA10 mutants (K→H, E, S, T, Y, N, Q, V, C, P, L, I, M, W, or F at position 2140) were unsuccessful because no CPE was observed in RD cells infected with each of these mutants, and no viral titers were detected. These results indicate that infectious CVA10 can be successfully rescued only when positively charged amino acids, Lys or Arg, are present at position 2,140. Therefore, only CVA10-K2140R mutant was selected for subsequent analysis.

To compare the growth kinetics of CVA10-WT (K2140) and CVA10-K2140R, RD cells were infected with each virus using a multiplicity of infection (MOI) of 0.01, and viral titers were measured at 0, 6, 24, and 48 h post-infection (hpi) by TCID_50_ method. As shown in Fig. 4B, CVA10-K2140R could replicate in RD cells, but it grew significantly worse than CVA10-WT at 24 and 48 hpi. These results suggest that the reduced infection phenotype of the CVA10-K2140R mutant is likely due to decreased KRM1-binding affinity.

Next, rescued CVA10-WT (K2140) and CVA10-K2140R viruses were tested for sensitivity to neutralization by hKRM1-Fc. Compared with CVA10-WT, CVA10-K2140R was resistant to neutralization with hKRM1-Fc (Fig. 4C), suggesting that there may be differences in KRM1 receptor protein binding between the two viruses. To test the idea, CVA10-WT and CVA10-K2140R viral particles were separately purified from 1L of infected RD cell culture supernatants and then compared for their KRM1 receptor-binding capacity by ELISA (Fig. 4D–F). Results of SDS-PAGE and electron microscopy analyses revealed that purified CVA10-WT and CVA10-K2140R viral particles were mature virions (full particles) consisting of VP1, VP2, and VP3 proteins (Fig. 4D and E; Fig. S2). Receptor binding ELISA analysis showed that CVA10-WT effectively reacted with hKRM1-Fc dose-dependently, whereas CVA10-K2140R showed very weak binding to hKRM1-Fc only at the highest virus concentration (2 µg/mL) (Fig. 4F). This finding was consistent with the results of hKRM1-Fc neutralization assay (Fig. 4C).

To compare the pathogenesis, 2-day-old ICR mice were inoculated with the same amount (particle number) of purified CVA10-WT or CVA10-K2140R. Mice were observed daily for survival and clinical signs after infection. As shown in Fig. 4G, CVA10-WT-infected mice began to develop clinical symptoms at 4 dpi and ultimately all died within 9 dpi. By contrast, only 50% of mice inoculated with CVA10-K2140R eventually died. There was a significant difference in the survival rate between the CVA10-WT and CVA10-K2140R groups. The reduced virulence phenotype of CVA10-K2140R mutant is likely due to a decrease in KRM1 receptor binding affinity.

### Residue K2140 is completely conserved in KRM1-dependent enteroviruses and is critical for their infection

**Fig 5:**
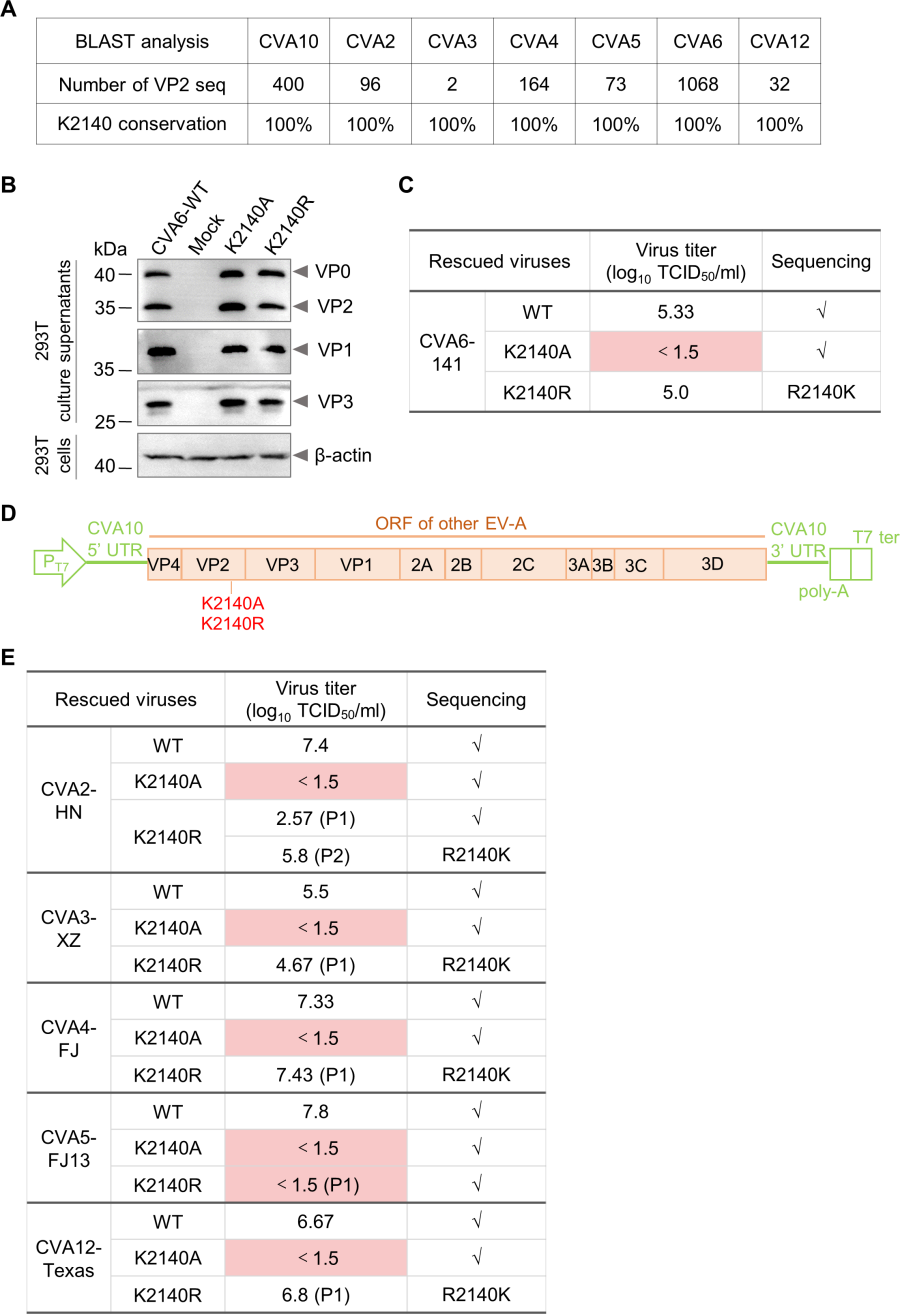
Residue K2140 is completely conserved in all KRM1-using enteroviruses and critical for their infection. (**A**) Conservation analysis of residue K2140 in KRM1-using enteroviruses. All VP2 protein sequences were obtained from NCBI database (as of October 2024). (**B**) Wild-type and mutant CVA6 infectious clones were transfected into 293T cells, and then, the culture supernatants were analyzed for viral capsid proteins by western blotting with anti-VP0, anti-VP1, and anti-VP3 polyclonal antibodies. Cell lysates were analyzed by western blotting with an anti-β-actin antibody. Mock, 293T cells transfected with the empty vector pVAX1 and the T7 RNA-pol plasmid. Note that 16 µL of 293T cell culture supernatants or lysates from 1  ×  10^5^ cells were boiled and loaded onto the gel. (**C**) The rescued wild-type and mutant CVA6 viruses were amplified in RD cells, titrated via TCID_50_ assay, and sequenced. (**D**) Schematic representation of the construction of chimeric infectious clones of CVA2, CVA3, CVA4, CVA5, and CVA12. Chimeric infectious clones were generated by replacing the open reading frames (ORF) in the CVA10 infectious clone plasmid with those from other enteroviruses. K2140A and K2140R mutations were separately introduced into these constructed infectious clones. (**E**) Wild-type and mutant CVA2, CVA3, CVA4, CVA5, and CVA12 viruses were rescued in transfected 293T cells and amplified in RD cells for one or two passages, followed by virus titration and Sanger sequencing. P1, passage 1; P2, passage 2. Note that the above experiments were performed twice, with similar results.

In addition to CVA10, several other enteroviruses, including CVA2, CVA3, CVA4, CVA5, CVA6, and CVA12, have been reported to use KRM1 protein as a functional cellular receptor (17). To assess the conservation of the K2140 residue among these enteroviruses, all VP2 protein sequences were downloaded from NCBI database (as of October 2024) and aligned. We found that residue K2140 was completely conserved in all of the KRM1-dependent enteroviruses (Fig. 5A).

We next investigated whether residue K2140 could play a key role in the infection of other KRM1-using enteroviruses besides CVA10. In recent years, CVA6 has become the dominant pathogen of HFMD outbreaks worldwide. Therefore, we first explored the role of residue K2140 in CVA6 infection by utilizing a previously reported infectious clone of CVA6 strain TW-141 (19). K2140A and K2140R mutations were separately introduced into the CVA6 infectious clone. Wild-type and mutant CVA6 infectious clones were transfected into 293T cells, and then, the culture supernatants were analyzed for viral capsid proteins by western blotting with CVA6 VP0-, VP1-, and VP3-specific antibodies. As shown in Fig. 5B and Fig. S3, CVA6 capsid protein bands were detected in the culture supernatants from 293T cells transfected with wild-type and mutant CVA6 infectious clones, suggesting the formation and secretion of CVA6 WT, K2140A, and K2140R viral particles. The rescued wild-type and mutant CVA6 viruses were amplified for one passage in RD cells, titrated by TCID_50_ assay, and sequenced. As shown in Fig. 5C, a high viral titer was detected for CVA6-WT, whereas no viral titer was detected for CVA6-K2140A mutant. In addition, the attempt to amplify CVA6-K2140R was unsuccessful because reverse mutation occurred in cell culture. The results suggest that the K2140 residue is essential for CVA6 infection of RD cells.

To assess the role of residue K2140 in CVA2, CVA3, CVA4, CVA5, and CVA12 infection, we synthesized the infectious clone plasmids of prototype strains of these viruses and attempted to rescue these viruses. CVA4 prototype strain High Point (CVA4-HP), and CVA12 prototype strain Texas-12 (CVA12-Texas) were successfully recovered after at least three serial passages on RD cells, although no obvious CPE was observed during the first passage. For CVA2 strain Fleetwood, CVA3 strain Olson, and CVA5 strain Swartz, no CPE was observed even after multiple passages on RD cells. Thus, we failed to rescue CVA2, CVA3, and CVA5.

To overcome this difficulty, we developed a new and efficient method to rescue enteroviruses by replacing the open reading frames (ORF) in the CVA10 infectious clone plasmid with those from other enteroviruses (Fig. 5D). ORF cDNA fragments were obtained by reverse transcription PCR (RT-PCR) from CVA2 strain HN, CVA3 strain XZ, CVA4 strain FJ, CVA5 strain FJ13, and CVA12 strain Texas. The chimeric CVA2-HN, CVA3-XZ, CVA4-FJ, CVA5-FJ13, and CVA12-Texas viruses were successfully rescued using this new strategy and produced obvious CPE on first passage in RD cells (Fig. 5E). K2140A and K2140R mutations were separately introduced into the chimeric infectious clones of these viruses. Mutant viruses were rescued in transfected 293T cells and amplified in RD cells for one or two passages, followed by titration via TCID_50_ assay and sequencing. As shown in Fig. 5E, for CVA2-K2140A, CVA3-K2140A, CVA4-K2140A, CVA5-K2140A, CVA5-K2140R, and CVA12-K2140A mutants, no viral titer was detected. For CVA2-K2140R, CVA3-K2140R, CVA4-K2140R, and CVA12-K2140R mutants, back mutations to wild-type occurred very rapidly. Taken together, these results demonstrate the importance of the Lys residue at position 2,140 in infection of all KRM1-dependent enteroviruses.

### CVA8 contains the conserved K2140 residue and uses KRM1 as a cellular receptor

**Fig 6:**
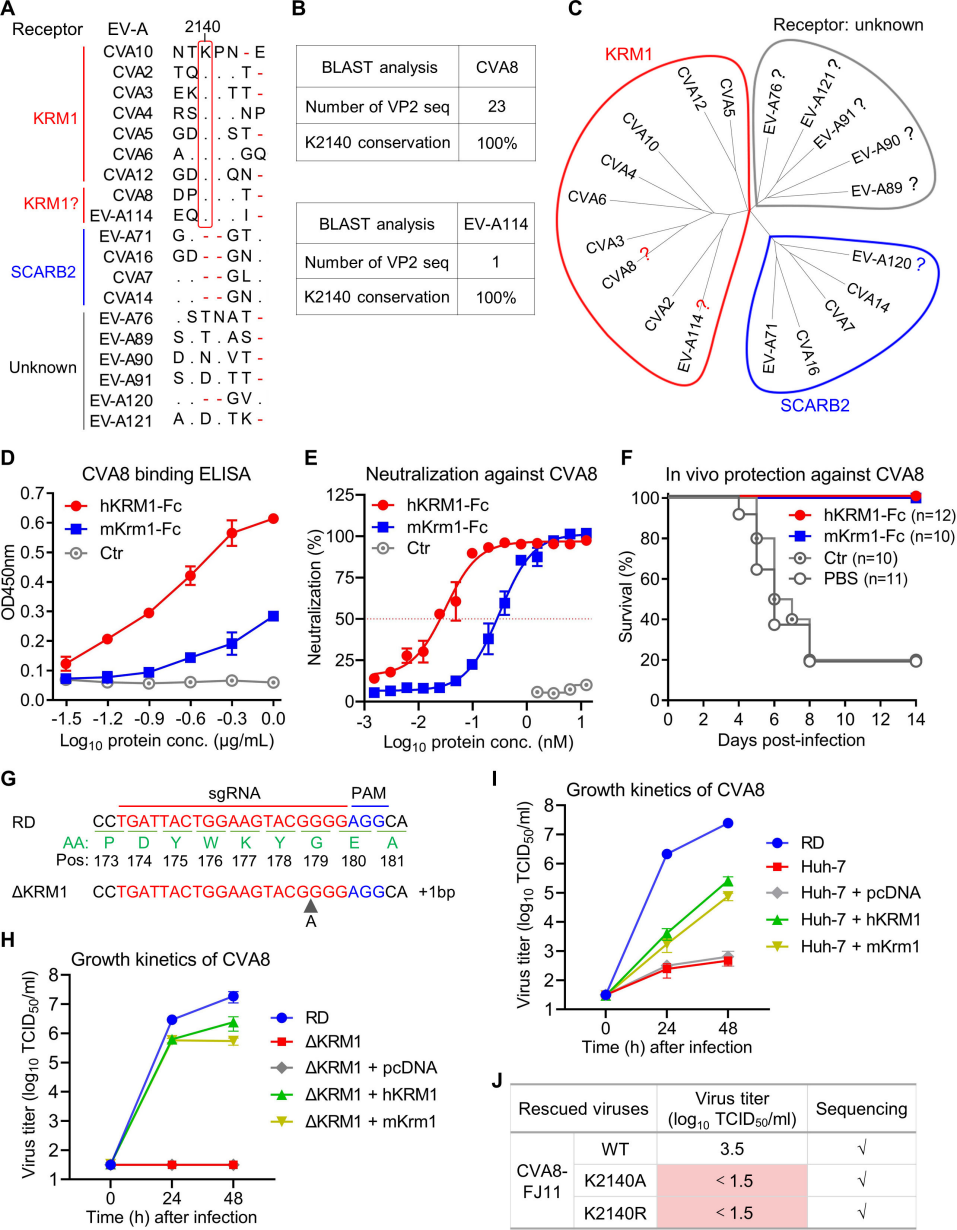
CVA8 contains the conserved K2140 residue and uses KRM1 as a cellular receptor. (**A**) Alignment (part) of amino acid sequences of VP2 EF loop of EV-A (prototype strains). Cellular receptors for EV-A are also shown. EV-A119 and simian enteroviruses are not included in the analysis. Dots represent residues identical to those of CVA10, and red dashes are gaps. The residue 2140 is boxed. (**B**) Conservation analysis of CVA8 and EV-A114 residue K2140. CVA8 and EV-A114 VP2 protein sequences were obtained from GenBank (as of October 2024). (**C**) Phylogenetic tree of EV-A constructed using VP2 protein sequences. Viruses using KRM1, SCARB2, and unknown receptors for infection are circled in red, blue, and gray, respectively. The question marks indicate that receptors for the indicated viruses have not yet been identified. (**D**) Reactivities of CVA8 with human and mouse KRM1-Fc protein were determined by ELISA. ELISA plates were coated with purified CVA8 (strain GS14) antigen and then incubated with serially diluted hKRM1-Fc and mKrm1-Fc protein. Ctr, ACE2-Fc. Data are means  ±  SEM of triplicate wells. (**E**) Neutralization of hKRM1-Fc and mKrm1-Fc protein against CVA8 strain GS14 was measured by cell viability assay. Ctr, ACE2-Fc. Data are means  ±  SEM of four biological replicate samples. (**F**) Soluble hKRM1-Fc and mKrm1-Fc proteins could inhibit CVA8 infection *in vivo*. 0.25 TCID_50_/mouse of CVA8 strain GS14 was incubated with PBS, ctr (ACE2-Fc), hKRM1-Fc or mKrm1-Fc for 1 h before intraperitoneal injection into 2-day-old ICR mice. After infection, survival and clinical signs were observed daily. Clinical scores were graded as described in the legend of Fig. 3. (**G**) Establishment of KRM1 knockout (ΔKRM1) RD cells by CRISPR/Cas9 system. The sgRNA targeting site and PAM site were shown in red and blue, respectively. The corresponding amino acid (AA) sequence was shown in green. Pos, amino acid position. Genomic sequence of KRM1 in KO cells was aligned with the wild-type sequence. (**H**) KRM1 is essential for CVA8 infection of RD cells. Wild-type RD cells, ΔKRM1 cells, ΔKRM1 cells mock-transfected with vector alone (pcDNA3.1), and ΔKRM1 cells transfected with cDNA of hKRM1 or mKrm1 were infected with CVA8 strain GS14 (MOI = 0.01) and viral titers were determined at 0, 24, or 48 h post-infection. (**I**) Overexpression of KRM1 in Huh7 cells conferred susceptibility to CVA8 infection. RD cells, Huh7 cells, Huh7 cells mock-transfected with vector alone (pcDNA3.1), and Huh7 cells transfected with hKRM1 or mKrm1 gene were infected with CVA8 strain GS14 (MOI = 0.01), and viral titers were determined at 0, 24, or 48 h post-infection. In panels H and I, data are means  ±  SD of triplicate biological samples. (**J**) Wild-type and mutant CVA8 viruses (strain FJ11) were rescued in transfected 293T cells and amplified in RD cells for one passage, followed by virus titration and sequencing. Note that the above experiments were repeated twice with similar results.

To analyze the conservation of the K2140 residue in other EV-A serotypes, sequence alignment of VP2 proteins from prototype strains of 19 serotypes within EV-A was performed, and the results showed that residue K2140 is also conserved in CVA8 and EV-A114 (Fig. 6A). Note that for EV-A114, only one VP2 protein sequence can be found in GenBank (as of October 2024). For CVA8, a total of 23 VP2 protein sequences were obtained from GenBank (as of October 2024), and residue K2140 was found to be completely conserved in all of them (Fig. 6B). To date, the cellular receptors for CVA8 and EV-A114 have not been identified. Phylogenetic analysis has proven to be an extremely valuable tool for predicting enterovirus receptor usage (20). The enteroviruses within the same evolutionary branch are highly likely to use the same cellular receptor (20). To predict the receptor usage of CVA8 and EV-A114, we performed a phylogenetic analysis of 19 EV-A serotypes based on VP2 sequence. The analysis revealed that EV-A serotypes diverged into three clusters. CVA8 and EV-A114 were closely related to the KRM1-dependent viruses CVA3 and CVA2, respectively (Fig. 6C). Thus, we speculated that CVA8 and EV-A114 may use KRM1 as a cellular receptor via capsid residue K2140. EV-A114 live virus was not available to us and therefore was not tested. Note that the genome sequence of EV-A114 strain deposited in GenBank was incomplete. Next, we tested whether the putative receptor for CVA8 was KRM1.

To determine whether CVA8 can bind directly to KRM1 protein, CVA8 binding ELISA assay was conducted (Fig. 6D). In this assay, ELISA plates were coated with purified CVA8 virion (strain GS14) and then incubated with serially diluted hKRM1-Fc, mKrm1-Fc protein, or control protein (ACE2-Fc). hKRM1-Fc and mKrm1-Fc, but not control protein ACE2-Fc, reacted with CVA8 virion. It is worth pointing out that hKRM1-Fc showed stronger binding to CVA8 virion than mKrm1-Fc. These results indicated that CVA8 can bind directly to both human and mouse KRM1.

We evaluated whether hKRM1-Fc could inhibit CVA8 infection *in vitro* (Fig. 6E). CVA8 were incubated with serially diluted hKRM1-Fc, mKrm1-Fc protein, or control protein (ACE2-Fc) before infection of RD cells. hKRM1-Fc and mKrm1-Fc, but not control protein ACE2-Fc, exhibited inhibitory effects in a protein dose-dependent manner. We should point out that hKRM1-Fc displayed more potent neutralizing activity (IC50 = 0.03 nM) against CVA8 than mKrm1-Fc (IC50 = 0.32 nM). We next explored whether hKRM1-Fc could inhibit CVA8 infection *in vivo* (Fig. 6F). CVA8 were incubated with PBS, hKRM1-Fc, mKrm1-Fc, or control protein (ACE2-Fc) for 1 h before infection of newborn ICR mice. Mice in the PBS and control groups became sick at 4 dpi and a large proportion (82%) of them eventually died. By contrast, all of the mice treated with hKRM1-Fc and mKrm1-Fc survived. These data demonstrate that soluble hKRM1 and mKrm1 proteins can effectively prevent CVA8 infection *in vitro* and *in vivo*.

Next, we tested whether CVA8 was dependent on KRM1 for infection. We first generated KRM1 knockout (ΔKRM1) RD cell clone by CRISPR/Cas9 system (Fig. 6G). Sanger sequencing showed that this ΔKRM1 cell clone carried a 1 bp insertion. Growth kinetics of CVA8 in different cells were compared (Fig. 6H). In this assay, wild-type RD cells, ΔKRM1 cells, ΔKRM1 cells mock-transfected with vector alone (pcDNA), and ΔKRM1 cells transfected with hKRM1 or mKrm1 cDNA construct for the overexpression of KRM1 were infected with CVA8, and viral titers were determined at 0, 24, or 48 hpi. No obvious viral replication was observed for ΔKRM1 cells and pcDNA-transfected ΔKRM1 cells. Exogenous expression of hKRM1 or mKrm1 genes was able to restore the susceptibility of ΔKRM1 cells to CVA8 infection. At 48 hpi, the viral titer in ΔKRM1 cells expressing hKRM1 cDNA was about 4.5-fold higher than that in ΔKRM1 cells expressing mKrm1 cDNA. These data demonstrate that KRM1 is essential for CVA8 infection of RD cells.

We found that the human hepatoblastoma-derived cell line Huh7 was resistant to CVA8 infection. To test whether overexpression of KRM1 could make Huh7 cells susceptible to CVA8, Huh7 cells were transfected with vector alone (pcDNA) or vectors containing hKRM1 or mKrm1 cDNA and then infected with CVA8 (Fig. 6I). Wild-type Huh7 and pcDNA-transfected Huh7 cells allowed only very inefficient CVA8 infection. Overexpression of hKRM1 or mKrm1 genes could confer the susceptibility of Huh7 cells to CVA8 infection. At 48 hpi, viral titers in Huh7 cells expressing hKRM1 or mKrm1 cDNA were about 406-fold and 116-fold higher than that in pcDNA-transfected Huh7 cells, respectively. These results demonstrate that KRM1 is sufficient to permit CVA8 infection of Huh7 cells.

To evaluate the role of residue K2140 in CVA8 infection, we constructed the infectious clone plasmids of CVA8 strains GS14 and FJ11 using the above chimeric strategy (Fig. 5D). The chimeric CVA8-FJ11, but not CVA8-GS14, were successfully rescued and produced obvious CPE on the first passage in RD cells (Fig. 6J). Next, the K2140A and K2140R mutations were separately introduced into the CVA8-FJ11 infectious clone. Mutant viruses were rescued in transfected 293T cells and amplified in RD cells for one passage, followed by titration and sequencing. These results showed that no viral titer was detected for CVA8-K2140A and CVA8-K2140R mutants (Fig. 6J). Thus, the K2140 residue is essential for CVA8 infection.

### Residue D90 of KRM1 interacts with residue K2140 of KRM1-using enteroviruses and plays an important role in enteroviral infection

**Fig 7:**
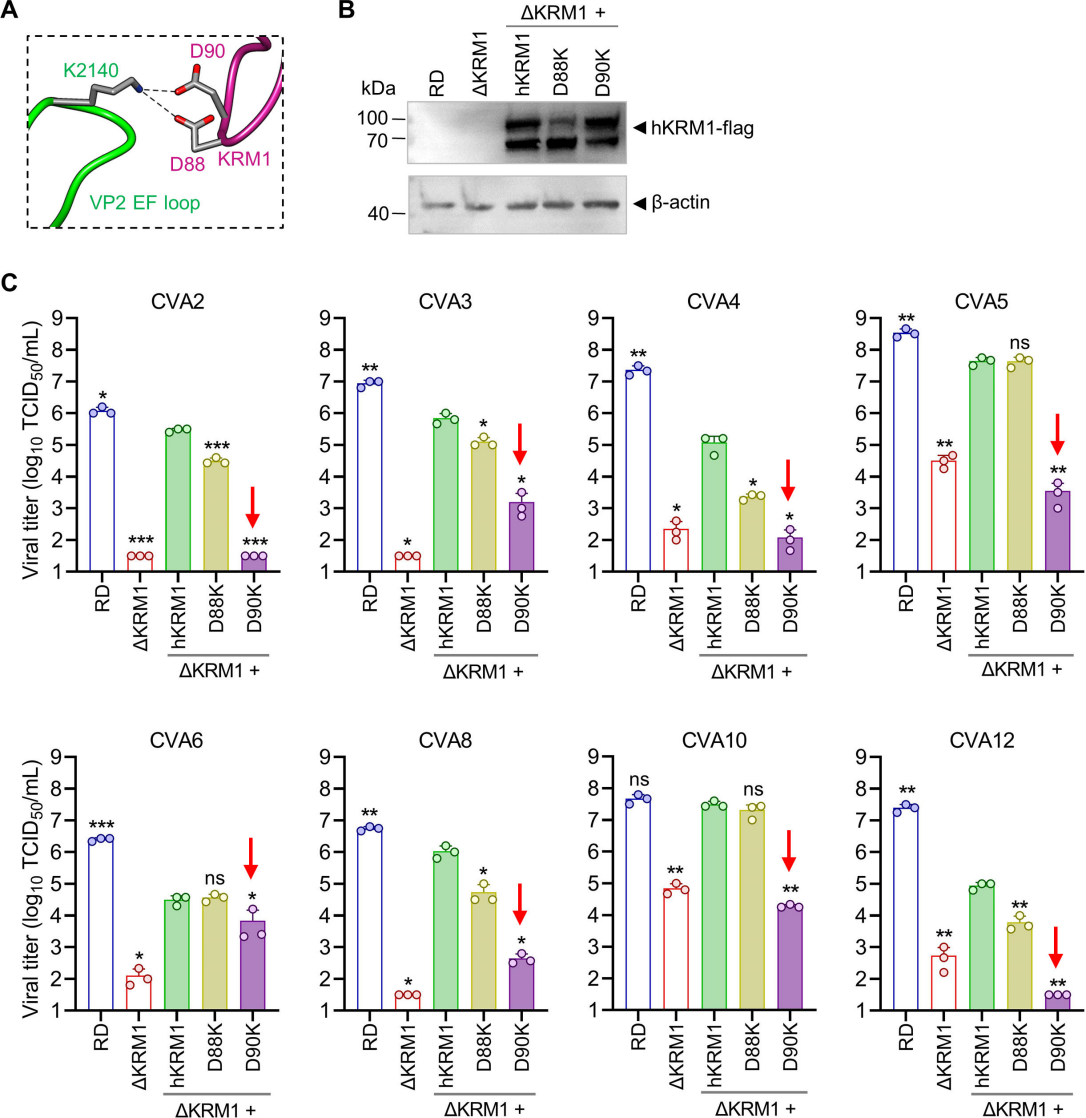
Residue D90 of KRM1 interacts with residue K2140 of KRM1-using enteroviruses and plays a key role in viral infection. (**A**) Residue K2140 of CVA10 forms hydrogen bonds and salt bridges with residues D88 and D90 of KRM1. VP2, green; KRM1, violet red. Nitrogen atom, blue; oxygen, red; and carbon, gray. Black dash lines indicate hydrogen bonds and salt bridges. (**B**) ΔKRM1 cells were transfected with wild-type or mutant hKRM1-flag cDNA and then subjected to western blotting analysis with anti-flag and anti-β-actin antibodies. Wild-type RD cells and non-treated ΔKRM1 cells were also analyzed by western blotting analysis. Lysates from 1  ×  10^5^ cells were boiled and loaded onto the gel. (**C**) Wild-type RD cells, ΔKRM1 cells, and ΔKRM1 cells expressing wild-type or mutant hKRM1-flag were infected with KRM1-using enteroviruses, and viral titers were measured at 24 hpi. Data are means  ±  SD of triplicate biological samples. Each data point represents a single biological replicate from a well in 24-well cell culture plates. The results were analyzed by two-tailed Student’s *t* test to determine the statistical significance between the hKRM1-complemented ΔKRM1 group and the other groups. ns, not significant; *, *P* < 0.05; **, *P* < 0.01; ***, *P* < 0.001. The downward arrows indicate that infectious viral titers in the ΔKRM1 + hKRM1-D90K cells were significantly lower than those in the hKRM1-complemented ΔKRM1 cells. Note that the above experiments were repeated at least twice with similar results.

According to the previously reported structure of CVA10 in complex with KRM1 receptor (PDB: 7BZU) (14), the side chain amino group of the K2140 residue of CVA10 forms hydrogen bonds and salt bridges with the side chain carboxyl group of residues D88 and D90 of KRM1 (Fig. 7A). We wanted to investigate whether residues D88 and D90 of KRM1 play a role in enterovirus infection. To answer this question, D88K and D90K mutations were separately introduced into hKRM1-flag cDNA. Wild-type RD cells, ΔKRM1 cells, and ΔKRM1 cells transfected with wild-type or mutant hKRM1-flag cDNA were infected with KRM1-using enteroviruses, and viral titers were determined at 24 hpi (Fig. 7B through C). Western blotting analysis with an anti-flag antibody showed that wild-type hKRM1, hKRM1-D88K, and hKRM1-D90K proteins were successfully expressed in the transfected ΔKRM1 cells (Fig. 7B; Fig. S4). As shown in Fig. 7C, infection assays showed that for CVA2, CVA3, CVA4, CVA5, CVA6, CVA8, CVA10, and CVA12, high viral titers were detected in wild-type RD cells, and a striking reduction in viral titers was observed in ΔKRM1 cells, confirming the conclusion of a previous study that these enteroviruses were dependent on KRM1 protein for efficient infection (17). In addition, exogenous expression of hKRM1 gene could restore the susceptibility of ΔKRM1 cells to infection with these viruses. Infectious viral titers in the ΔKRM1 cells expressing the hKRM1-D88K mutant were slightly lower than or comparable with those in the ΔKRM1 cells expressing wild-type hKRM1. By contrast, infectious titers of each tested enterovirus in the ΔKRM1 cells expressing the hKRM1-D90K mutant were significantly lower than those in the wild-type hKRM1-complemented ΔKRM1 cells. These results strongly suggest that residue D90 of KRM1 could play a vital role in KRM1-dependent enterovirus infection.

## DISCUSSION

In this study, we identified K2140 as a critical residue for CVA10 infection by scanning mutagenesis of KRM1 receptor-contacting residues. Residue K2140 plays a vital role not only in receptor recognition, cell attachment, and infection of CVA10 but also in CVA10 pathogenicity in mice. More importantly, residue K2140 is completely conserved in all of the previously reported KRM1-dependent enteroviruses and is essential for viral infection. Moreover, another enterovirus, CVA8, was found to contain the conserved K2140 residue and be able to use KRM1 as a cellular receptor. In addition, residue D90 of KRM1 interacts with residue K2140 of KRM1-using enteroviruses and plays a key role in their infection.

KRM1 protein can function as a cellular receptor for a major group of enteroviruses, including CVA2, CVA3, CVA4, CVA5, CVA6, CVA10, and CVA12 (17). The molecular basis for the broad recognition of these viruses by KRM1 receptor still remains unclear. In this study, we found that residue K2140 is 100% conserved among all KRM1-dependent enteroviruses (Fig. 5A). This conserved residue forms electrostatic and hydrogen-bonding interactions with residue D90 of KRM1, which is vital for virus-receptor binding and subsequent infection (Fig. 2 to 5 and 7). Our findings offer insights into how KRM1 can recognize multiple enteroviral serotypes. Undoubtedly, there are other key residues and molecular mechanisms involved in KRM1 receptor recognition of these enteroviruses. More studies are currently underway.

Previous studies reported that KRM1 receptor binds to the canyon region of CVA10 (12, 14). Residue K2140 is located at the south rim of the canyon and is highly surface-exposed (Fig. 1B and C). Thus, K2140 must undergo a high immune pressure. Under immune pressure, enteroviruses usually accumulate mutations that facilitate immune evasion. However, NCBI BLAST analysis revealed that residue K2140 is completely conserved among all KRM1-dependent enteroviruses, and no naturally occurring mutations at position 2,140 were found (Fig. 5A). In line with the conservation analysis, the saturation-mutagenesis analysis showed that alterations at residue K2140 could either abolish or drastically diminish the infectivity of CVA10 (Fig. 4). Similar results were observed upon mutation of the K2140 residue in other KRM1-dependent enteroviruses (Fig. 5). Thus, mutations at residue K2140 are detrimental to viral adsorption and infection of KRM1-using enteroviruses (Fig. 2, 4, and 5), highlighting the importance of the Lys residue at position 2,140 in viral infection. These findings may contribute to the development of antiviral drugs with broad-spectrum activity against all KRM1-dependent enteroviruses.

We previously demonstrated that VP2 peptide P28 (residues 136–150 of VP2) represents a CVA10-specific linear neutralizing and protective antigenic site (21). We also demonstrated that N2142, a residue within the P28 peptide region, is a critical part of the epitopes recognized by anti-CVA10 polyclonal antibodies and neutralizing MAb 2A11 (18). In this study, we found that mutation of K2140 (a residue within the P28 region) could render CVA10 resistant to the binding activity of anti-CVA10 neutralizing MAb 1B10 (Fig. 2F), suggesting that K2140 residue is a critical part of the 1B10 epitope. These findings demonstrate the important role of the P28 region in mediating CVA10-induced neutralizing antibody production. In addition, the VP2 P28 region was also identified as the neutralizing antigenic sites of other enteroviruses, including EV-A71 and EV-D68 (22–24).

Overall, our study reveals that VP2 residue K140 is absolutely conserved among all KRM1-dependent enteroviruses and is crucial for virus-receptor interactions and viral infection. These findings provide a deeper understanding of the molecular basis of enteroviral infection *in vitro* and *in vivo* and also offer valuable information for the development of broad-spectrum anti-enteroviral drugs.

## MATERIALS AND METHODS

### Cells and viruses

Human rhabdomyosarcoma (RD), HEK 293T, and human hepatocellular carcinoma Huh-7 cells were maintained in DMEM (Gibco, Thermo Fisher Scientific, USA) with 10% fetal bovine serum (FBS) at 37°C.

CVA10 prototype strain Kowalik (GenBank ID: AY421767), CVA2 strain HN202009 (HN; GenBank ID: MT992622) (25, 26), CVA3 strain XZ99086-1999 (XZ; National Microbiology Data Center [NMDC] ID: NMDCN0003ADI), CVA5 strain HN (GenBank ID: PP421226), and CVA8 strain 14-943/GS/CHN/2014 (GS14; ID: MT648781) (27, 28) were propagated in RD cells. CVA4 strain High Point (HP; ID: AY421762), CVA6 strain TW-2007-00141 (141; ID: KR706309) (19), and CVA12 strain Texas-12 (Texas; ID: AY421768) were successfully rescued from infectious clones. Viral titers were determined by the 50% tissue culture infectious dose (TCID_50_) assay on RD cells.

### Proteins and antibodies

His-tagged hKRM1-Fc and mKrm1-Fc fusion proteins consist of the ectodomain (residues A23 to G373) of human and mouse KRM1 and C-terminal human IgG1 Fc were successfully purified from HEK 293 F cells (18).

Anti-CVA10 sera were prepared by immunization of BALB/c mice with purified CVA10/Kowalik formulated with alum adjuvant (18). MAb 1B10 is a mouse CVA10-neutralizing IgG antibody, which was generated in our lab according to previously described methods (24, 29).

### Structural analysis of the interaction between CVA10 and KRM1

The cryo-EM structure of the CVA10-KRM1 complex (PDB: 7BZU) at pH 5.5 (14) was used for structural analysis. The structural representations were generated using UCSF Chimera software (v1.15) (30).

### Recovery and characterization of wild-type and mutant enteroviruses

The plasmid pVAX-CVA10-WT containing full-length cDNA of CVA10/Kowalik was constructed in a previous work (18) and used as a positive control. Mutant CVA10 cDNA clones were constructed by the introduction of single point mutations into pVAX-CVA10-WT using NEBuilder HiFi DNA Assembly Master Mix (NEB) and confirmed by sequencing the entire coding region. The CVA6 infectious clone plasmid pSVA-CVA6-141 was kindly provided by Dr. Tong Cheng, and mutant CVA6 clones were constructed as described above.

To construct CVA2, CVA3, CVA4, CVA5, CVA8, and CVA12 infectious clones, the ORF cDNA fragments were obtained by RT-PCR from CVA2 strain HN, CVA3 strain XZ, CVA4 strain FJ (NMDC ID: NMDCN0005SQA), CVA5 strain FJ13 (NMDC ID: NMDCN0005SQB), CVA8 strain FJ11 (NMDC ID: NMDCN0005SQC), and CVA12 strain Texas, respectively, and used to replace CVA10 ORF in the CVA10 infectious clone plasmid. Single point mutations were then introduced into CVA2, CVA3, CVA4, CVA5, CVA8, and CVA12 infectious clones, yielding mutant cDNA clones.

To rescue viruses, HEK 293T cells were seeded at a density of 2  ×  10^5^ cells/well into 24-well plates and incubated for 24  h. For the transfection, 200 ng of the infectious clone plasmid and 300 ng of the optimized T7 RNA polymerase-encoding plasmid was diluted in 150 µL Opti-MEM medium (Invitrogen, USA) without serum. In total, 1.5 µL Lipofectamine 2000 (Invitrogen) was diluted in 150 µL Opti-MEM medium. The diluted plasmids and Lipofectamine 2000 were mixed and incubated for 30 min at room temperature. The transfection mixture was added to each well and incubated for 6  h. The medium was replaced with DMEM (Gibco) with 1% FBS, and the cultures were maintained at 37°C. Three days post-transfection, the rescued viruses were harvested by freeze-thawing and propagated in RD cells in 24-well plates with DMEM and 1% FBS. Three days after infection, the viruses were collected, confirmed by sequencing of the RT-PCR products, and titrated by TCID_50_ assay.

### Viral neutralization assay

Fifty microliters/well of serially diluted KRM1-Fc protein was incubated with 100 TCID_50_/well of wild-type and mutant viruses for 1 h at 37°C in 96-well plates. Next, RD cells, at 20,000 cells per well, were added to the 96-well plates. Cells were cultured for 3 days before the examination of CPE. Neutralization concentration is defined as the lowest concentration of KRM1-Fc that is able to completely block CPE.

To determine the neutralizing potency of KRM1-Fc, a cell viability assay was performed with the CellCounting-Lite 2.0 Luminescent Cell Viability Assay kit (Vazyme, China) according to the manufacturer’s protocol. Percent neutralization was calculated is calculated from the following formula: 100 × (relative luminescence unit [RLU] of the sample − RLU of virus control)/(RLU of untreated cell control − RLU of virus control). IC50 values were calculated using Graphpad Prism (v8.0).

### Western blot analysis

The samples (cells, 293T culture supernatants, or sucrose gradient fractions) were boiled for 10 min in SDS loading buffer, separated by SDS-PAGE, and transferred onto PVDF membranes. The membranes were probed with horseradish peroxidase (HRP)-conjugated anti-β-actin antibody (Proteintech, China), HRP-conjugated anti-flag antibody (Proteintech), or virus-specific anti-VP0, anti-VP1, and anti-VP3 antibodies (31, 32), followed by a secondary antibody conjugated to HRP (Proteintech). Protein bands were visualized using the ECL western blotting substrate (Merck).

### Virus purification

To rescue CVA10-WT or CVA10-K2140A, 293T cells grown in 15 cm dishes were transfected with infectious clone plasmids and the T7 RNA-pol expression plasmid using polyethylenimine (PEI) max 40K (PolySciences, USA). 293T culture supernatants were collected at 72 h post-transfection and were precipitated with 10% polyethylene glycol (PEG) 8000 and 200 mM NaCl at 4°C overnight. The precipitate was collected by centrifugation and resuspended in 0.15 M PBS buffer. After centrifugation to remove impurities, the samples were layered on top of a 20% sucrose cushion and ultra-centrifuged at 27,000 rpm for 4 h. Resulting pellets were resuspended in PBS. After a centrifugation step for clarification, the virus samples were further purified by ultra-centrifugation through a 10%–50% sucrose gradient at 39,000 rpm for 3 h. The resulting gradient fractions were analyzed by SDS-PAGE. Protein concentrations were measured by Bradford assay (Bio-Rad).

To purify CVA10-WT, CVA10-K2140R, and CVA8, RD cells were infected at a MOI of 0.01 and cultured for 3 days. Viral particles in the culture supernatants were purified according to the above procedure (PEG precipitation and ultra-centrifugation).

### Electron microscopy analysis

Viral samples were diluted to 50 µg/mL in PBS and dropped onto glow-discharged carbon-coated grids. Samples were negatively stained with uranyl acetate and imaged in a Tecnai G2 Spirit transmission electron microscope (FEI, USA) operated at 200 kV.

### Antibody-binding ELISA

ELISA plates were coated with serially diluted purified CVA10 viral particles at 4°C overnight. After blocking with 5% milk in PBST, the plates were incubated with 1:10,000 diluted anti-CVA10 sera or 100 ng/well of MAb 1B10 at 37°C for 2 h. After washing, the plates were incubated with HRP-conjugated anti-mouse IgG (Proteintech) at 37°C for 1 h. Absorbance was read at 450 nm after color development.

### Virus attachment assay

Five hundred microliters/well of purified CVA10 viral particles (0.1 µg/mL) was added to cooled RD cells plated in 24-well plates and incubated at 4°C for 2 h to allow attachment. Then, cold PBS was used to wash the unbound viral particles. Afterward, the total RNA from cells was extracted using TRNzol reagent (TIANGEN, China). cDNA was transcripted using HiScript Ill 1st Strand cDNA Synthesis Kit (Vazyme, China). Quantitative PCR was performed using SYBR Premix Ex Taq kit (Takara), and relative CVA10 mRNA expression levels were normalized to that of β-actin. CVA10-specific primers were as follows: forward primer, 5′-GAAATGGAGTGTTGGAGGCGA-3′; reverse primer, 5′- TTTCTGTTGTAGTGACGAATG-3′. β-actin primers were as follows: forward primer, 5′-GGACTTCGAGCAAGAGATGG-3′; reverse primer, 5′-AGCACTGTGTTGGCGTACAG-3′.

### Receptor-binding ELISA

To compare receptor-binding activities of CVA10-WT and CVA10 mutants, ELISA plates were coated with serially diluted purified CVA10 viral particles at 4°C overnight. After blocking with 5% milk, the plates were incubated with 100 ng/well of hKRM1-Fc for 1 h at room temperature. After washes, the plates were incubated with HRP-conjugated anti-human IgG (Proteintech) for 1 h at room temperature. Absorbance was read at 450 nm after color development.

To compare CVA8-binding activities of human and mouse KRM1 protein, ELISA plates were coated with 100 ng/well of purified CVA8 viral particles at 4°C overnight. After blocking with 5% milk, the plates were incubated with serially diluted hKRM1-Fc, mKrm1-Fc protein, or control protein (ACE2-Fc) (33) at room temperature for 1 h. After washing, the plates were incubated with HRP-conjugated anti-human IgG at room temperature for 1 h. Absorbance was read at 450 nm after color development.

### *In vivo* infection assay

To compare *in vivo* virulence of CVA10-WT or CVA10-K2140A, groups of 2-day-old ICR mice were inoculated intraperitoneally (i.p.) with PBS or 10^−5^ ng/mouse of purified CVA10-WT or CVA10-K2140A. Following infection, mice were observed daily for clinical symptoms and mortality for 14 days. Clinical scores were graded as follows: 0, healthy; 1, reduced mobility; 2, limb weakness; 3, paralysis; and 4, death. To measure viral loads in organs, groups of infected mice were euthanized at 4 dpi, and limb muscle and spinal cord tissues were harvested, weighed, and homogenized in 400 µL of Trizol for the analysis of viral RNA levels by RT-qPCR. The CVA10 infectious clone plasmid was used as a standard to determine the absolute viral genome copy numbers. For pathological analysis, groups of infected mice were euthanized at 4 dpi, and the limb muscles were collected, fixed in 4% paraformaldehyde, and stained with hematoxylin and eosin (H&E) (Servicebio, China), followed by observation under a light microscope.

To compare *in vivo* virulence of CVA10-WT or CVA10-K2140R, groups of 2-day-old ICR mice were injected i.p. with 1 ng/mouse of purified CVA10-WT or CVA10-K2140R. Survival and clinical symptoms were monitored daily for 14 days.

To assess whether KRM1 protein could inhibit CVA8 infection *in vivo*, 0.25 TCID_50_/mouse of CVA8 were incubated with PBS, 10 µg/mouse of hKRM1-Fc, mKrm1-Fc, or control protein (ACE2-Fc) (33) at room temperature for 1 h. The mixtures were separately injected i.p. into 2-day-old ICR mice (≥10 mice/group). Survival and clinical symptoms were monitored daily for 14 days.

### Viral growth kinetics

Before the infection assay, human and mouse KRM1 coding sequences were cloned into pcDNA3.1 vector with 3× flag tag at the C-terminus. D88K and D90K mutations were separately introduced into the pcDNA3.1-hKRM1-flag vector, yielding mutant hKRM1 cDNA plasmids. Cells (RD, RD-ΔKRM1, transfected ΔKRM1, Huh7, or transfected Huh7 cells) in 24-well plates were infected with the indicated viruses at a MOI of 0.01 at 37°C for 1 h. The cells were washed with PBS and incubated in fresh medium at 37°C for different times. Subsequently, the cells and culture supernatants were subjected to freeze-thawing, and viral titers were measured by TCID_50_ method.

### Sequence conservation analysis

VP2 protein sequences of EV-A were downloaded from the NCBI database (as of October 2024) by BLAST and aligned using CLC Sequence Viewer software (v8.0) for conservation analysis. Phylogenetic tree construction was performed using CLC Sequence Viewer software (v8.0) with the neighbor-joining method, and a bootstrap analysis was performed for 100 replicates.

### Generation of ΔKRM1 cell line by CRISPR/Cas9 system

The single-guide RNA (sgRNA) targeting exon 5 of the hKRM1 gene (5′-TGATTACTGGAAGTACGGGG-3′) was designed and synthesized. The sgRNA and Cas9 proteins were incubated and co-transfected into RD cells by electroporation. Single-cell ΔKRM1 clones were screened and validated by CVA10 infection assay. Next, genomic DNA was extracted, and the exon 5 region was amplified by PCR and cloned into a pMD19-T vector (Takara) for Sanger sequencing.

## Data Availability

All relevant data are provided as figures within the paper. More detailed methods are available from the corresponding author upon request.

## SUPPLEMENTAL MATERIAL

Fig. S1 to S4 mbio.03040-24-s0001.pdf Raw images of western blot and SDS-PAGE.

ASM does not own the copyrights to Supplemental Material that may be linked to, or accessed through, an article. The authors have granted ASM a non-exclusive, world-wide license to publish the Supplemental Material files. Please contact the corresponding author directly for reuse.
