# Supplementary material for: Completely conserved VP2 residue K140 of KREMEN1-dependent enteroviruses is critical for virus-receptor interactions and viral infection
Source: mBio. 2025 Jan 16;16(2):e03040-24. doi: 10.1128/mbio.03040-24 (PMC11796367; doi:10.1128/mbio.03040-24)
Supplement: Fig. S1 to S4 — Raw images of western blot and SDS-PAGE. [file mbio.03040-24-s0001.pdf]

<u>**Supplementary Information**</u>

Completely conserved VP2 residue K140 of KREMEN1-dependent enteroviruses is critical for virus-receptor interactions and viral infection

by Zeyu Liu *et al*.

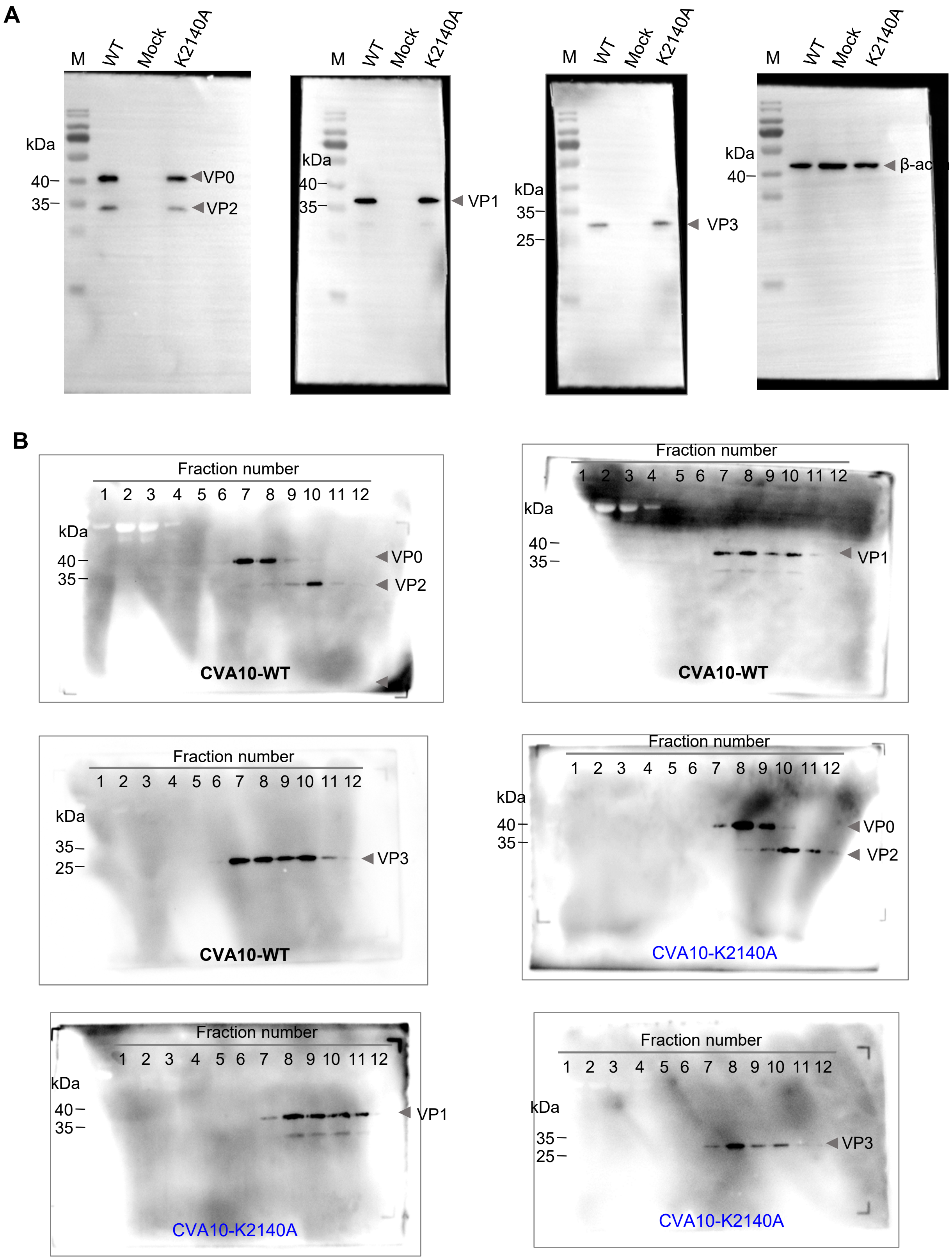

Figure S1. Raw images of western blot of Fig. 2B (A) and Fig. 2C (B).

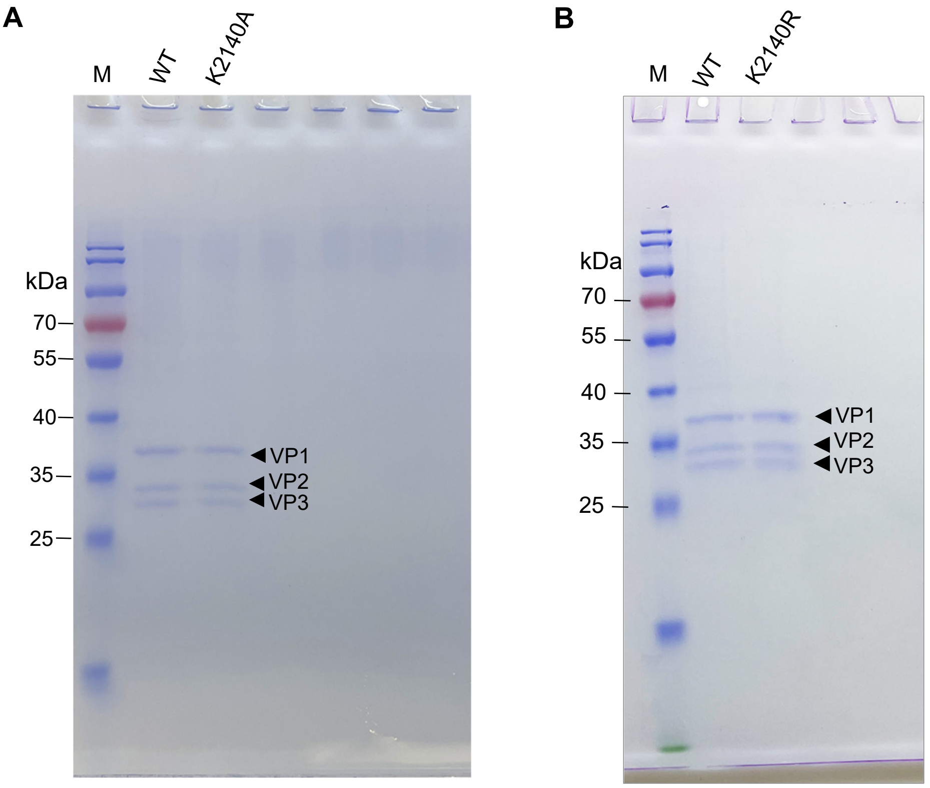

Figure S2. Raw images of SDS-PAGE of Fig. 2D (A) and Fig. 4D (B).

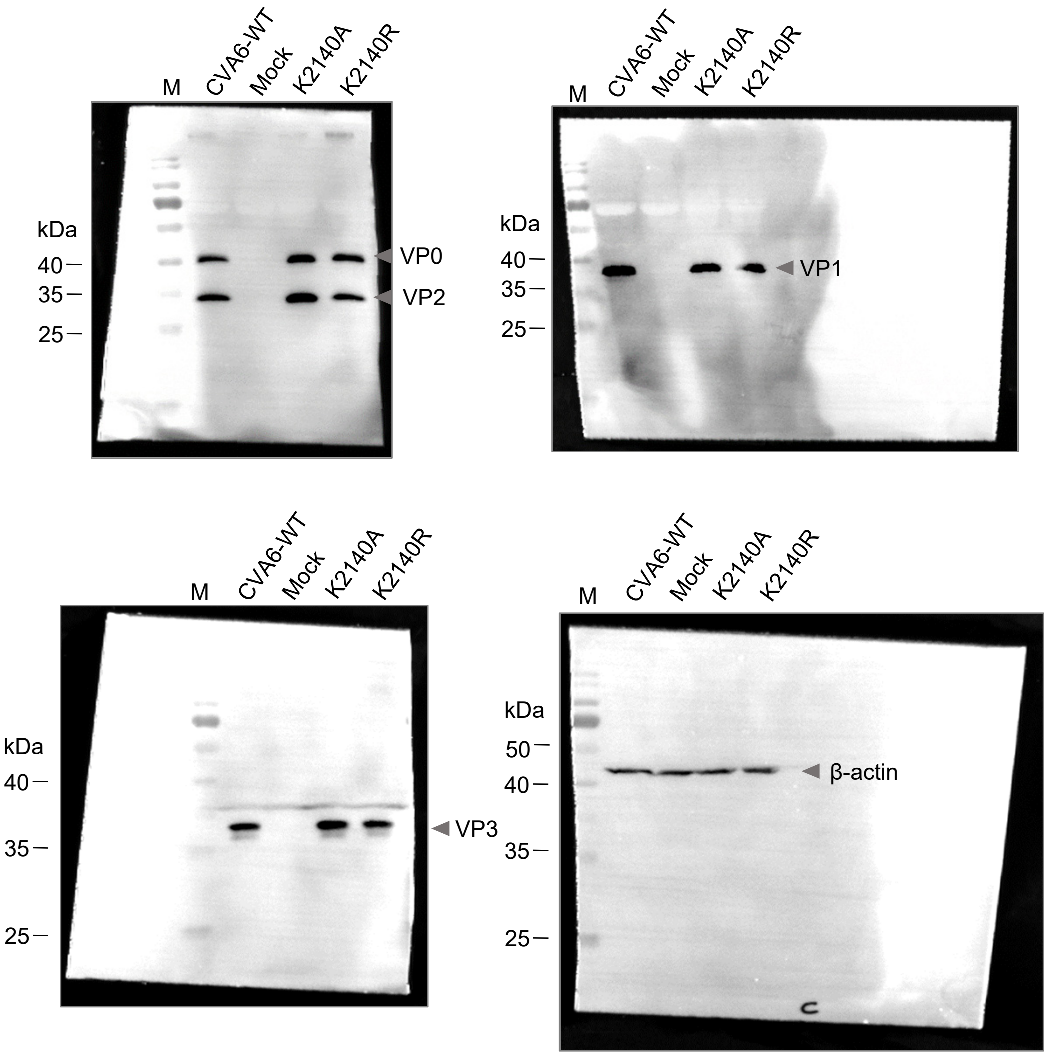

Figure S3. Raw images of western blot of Fig. 5B.

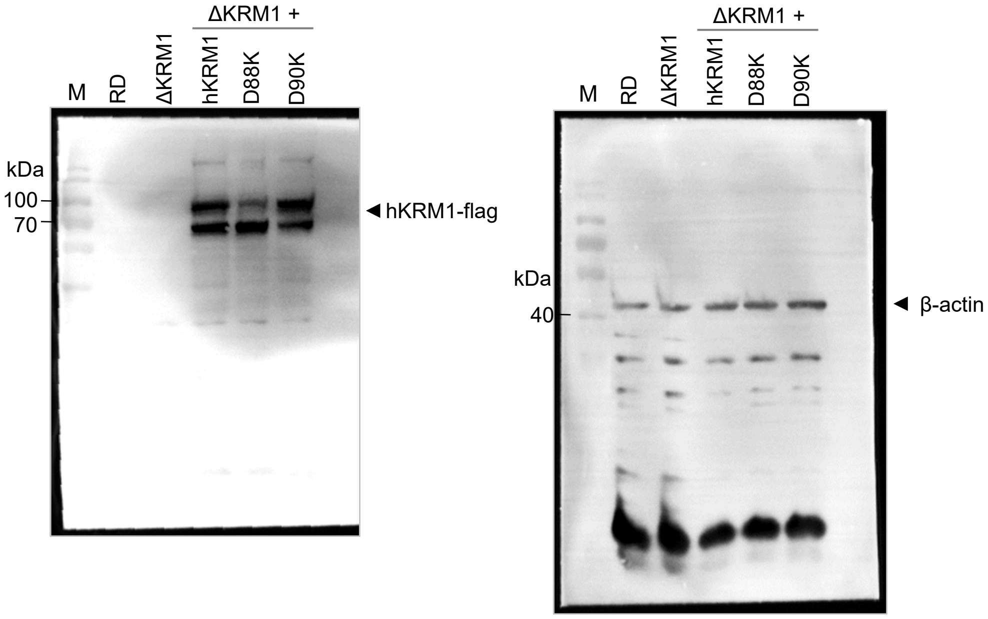

Figure S4. Raw images of western blot of Fig. 7B.
